# Novel Bioassay for the Discovery of Inhibitors of the 2-*C*-Methyl-*D*-erythritol 4-Phosphate (MEP) and Terpenoid Pathways Leading to Carotenoid Biosynthesis

**DOI:** 10.1371/journal.pone.0103704

**Published:** 2014-07-31

**Authors:** Natália Corniani, Edivaldo D. Velini, Ferdinando M. L. Silva, N. P. Dhammika Nanayakkara, Matthias Witschel, Franck E. Dayan

**Affiliations:** 1 São Paulo State University, Faculty of Agronomic Sciences, Botucatu, SP, Brazil; 2 National Center for Natural Products Research, School of Pharmacy, University of Mississippi, University, MS, United States of America; 3 BASF SE, GVA/HC-B009, Ludwigshafen, Germany; 4 USDA-ARS Natural Products Utilization Research Unit, University, MS, United States of America; Albert-Ludwigs-University, Germany

## Abstract

The 2-*C*-methyl-*d*-erythritol 4-phosphate (MEP) pathway leads to the synthesis of isopentenyl diphosphate in plastids. It is a major branch point providing precursors for the synthesis of carotenoids, tocopherols, plastoquinone and the phytyl chain of chlorophylls, as well as the hormones abscisic acid and gibberellins. Consequently, disruption of this pathway is harmful to plants. We developed an *in vivo* bioassay that can measure the carbon flow through the carotenoid pathway. Leaf cuttings are incubated in the presence of a phytoene desaturase inhibitor to induce phytoene accumulation. Any compound reducing the level of phytoene accumulation is likely to interfere with either one of the steps in the MEP pathway or the synthesis of geranylgeranyl diphosphate. This concept was tested with known inhibitors of steps of the MEP pathway. The specificity of this *in vivo* bioassay was also verified by testing representative herbicides known to target processes outside of the MEP and carotenoid pathways. This assay enables the rapid screen of new inhibitors of enzymes preceding the synthesis of phytoene, though there are some limitations related to the non-specific effect of some inhibitors on this assay.

## Introduction

The terms isoprenoid, terpenoid, and terpene are used interchangeably in the literature to refer to a broad class of natural products derived from C5 isopentenyl diphosphate (IPP) [1], [2]. Plants produce a myriad of isoprenoids that are functionally important in many physiological and biochemical processes [3], [4]. Carotenoids comprise a large isoprenoid family that are derived from the C40 tetraterpenoid phytoene [5] and produced by all photosynthetic organisms (plants, algae and cyanobacteria) as well as certain non-photosynthetic bacteria and fungi [6]. In plants, carotenoids participate in photosynthetic processes, including light harvesting, energy conversion, electron transfer, and quenching of excited chlorophyll triplets [7] in addition to a number of other functions.

Through evolution, two independent biosynthetic routes have been selected for the synthesis of these two basic building blocks [8]. In the cytosol and mitochondria, IPP and dimethylallyl diphosphate (DMAPP) are assembled from three molecules of acetyl-CoA by the mevalonate (MVA) pathway. This pathway was first described in the early work of Bloch and Lynen [9], [10], and was thought to be the sole source of all terpenoids. However, it is now known that it is responsible for the synthesis of sterols and ubiquinone. The MVA pathway is the subject of several reviews [5], [11], and is not the focus of this paper.

**Figure 1:**
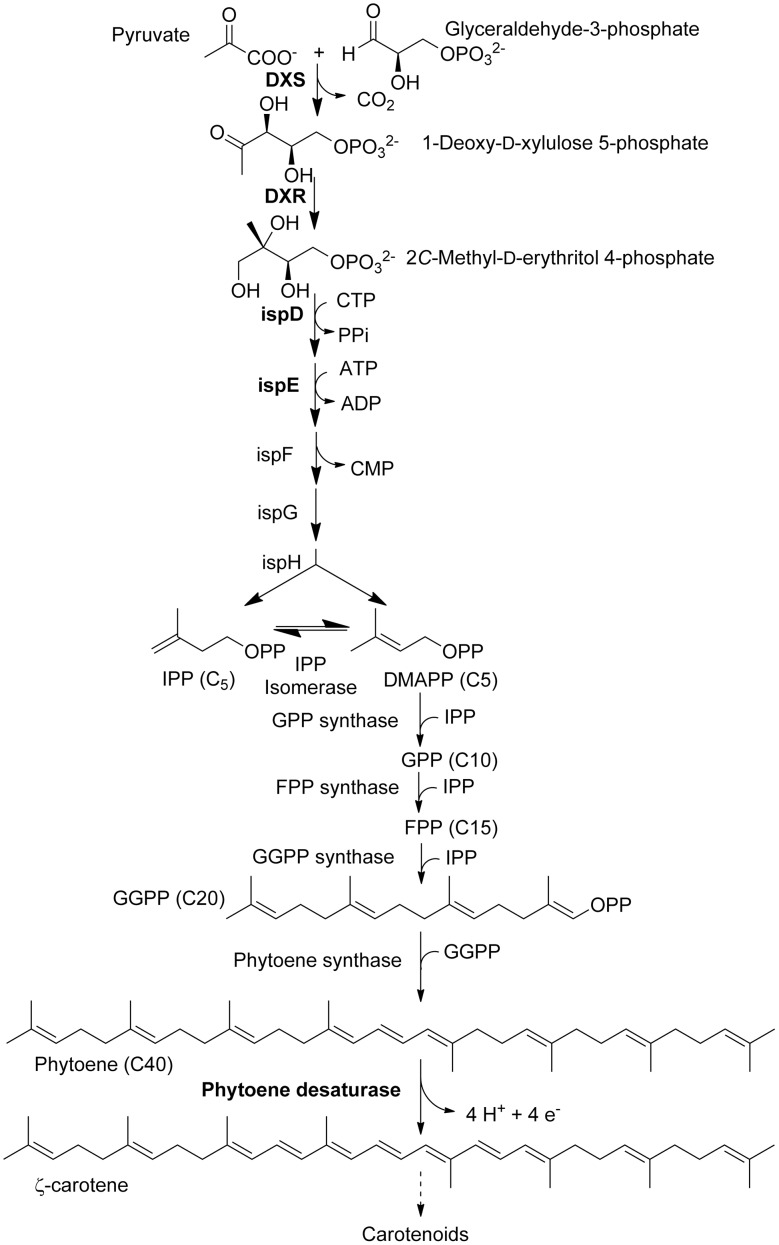
Biosynthesis of carotenoids starts with the 2-*C*-methyl-*d*-erythritol 4-phosphate (MEP) pathway leading to the formation of IPP, continues with the isoprenoid pathway to obtain GGPP. The first committed step to the synthesis of carotenoids consists of the head to head condensation of 2 GGPP to form phytoene. The enzymes in bold letters denote enzyme targets that were tested in this study.

The existence of an alternative pathway was suggested based on the observation that genes encoding enzymes catalyzing the late steps of the MVA pathway are absent in some archaeal genomes [12]. Furthermore, plants treated with the herbicide clomazone had reduced carotenoid levels but their levels of sterols were not affected [13]–[15]. This plastid-localized independent pathway, called the 2-*C*-methyl-*d*-erythritol 4-phosphate (MEP) pathway (also non-mevalonate or 1-deoxy-*d*-xylulose 5-phosphate (DOXP) pathway), was reported in 1997 (Figure 1) [9], [16]–[19]. The MEP pathway is a major branch point providing precursors for the synthesis of plastidic monoterpenes, diterpenes, carotenoids, the phytyl chain of chlorophylls, tocopherols, plastoquinone as well as the hormones abscisic acid and gibberellins. There is limited crossover between the two pathways [20], [21].

The MEP and carotenoid pathways are well characterized and have been reviewed extensively [1], [5], [12], [22]. Briefly, carotenoid biosynthesis can be divided into three phases (Figure 1). Phase I includes the formation of IPP and DMAPP via the plastid-localized MEP pathway. The first step of the MEP pathway is catalyzed by 1-deoxy-*d*-xylulose 5-phosphate synthase (DXS, EC 2.2.1.7), converting pyruvate and glyceraldehyde-3-phosphate to 1-deoxy-*d*-xylulose 5-phosphate (DOXP). The intramolecular rearrangement and reduction of DOXP to 2-*C*-methyl-*d*-erythritol 4-phosphate (MEP) is catalyzed by 1-deoxy-*d*-xylulose 5-phosphate reductoisomerase (DXR, EC 1.1.1.267). Diverse experimental evidence demonstrates that DXS and DXR represent potential regulatory control points in the MEP pathway [23], [24]. Phase I concludes with the formation of the C5 building blocks IPP and DMAPP. In Phase II, a single DMAPP serves as the substrate for successive head-to-tail condensations of IPP units to ultimately form the C20 geranylgeranyl diphosphate (GGPP) [25]. Phase III begins with the head to head condensation of two GGPP molecules to produce phytoene (Figure 1) by the enzyme phytoene synthase (PSY, EC 2.5.1.32). Subsequently, phytoene desaturase (PDS, EC 1.3.5.5) and **ζ**-carotene desaturase (ZDS, EC 1.3.5.6) catalyze similar dehydrogenation reactions introducing four double bonds in phytoene to form lycopene. Desaturation requires a plastid-localized terminal oxidase and plastoquinone in photosynthetic tissues [26], [27].

Carotenoids are important for plant survival, especially in their role as protection from photooxidation [4], [28]. Several important bleaching herbicides inhibit carotenoid synthesis [29]. PDS is the target site for several herbicides such as norflurazon, fluridone and flurochloridone [30]. When sensitive plants are exposed to these herbicides, PDS activity is inhibited, resulting in a rapid accumulation of phytoene and cessation of carotenoid biosynthesis [31]. In plants, inhibition of *p*-hydroxyphenylpyruvate dioxygenase (HPPD, EC 1.13.11.27) by triketone, isoxazole and pyrazole herbicides affects the formation of homogentisic acid [32], which is a key precursor for the biosynthesis of plastoquinone, a critical co-factor of PDS [26] and leads to inhibition of its enzymatic activity. This class of herbicides represents the last herbicide mode of action to have been commercialized in the last twenty years [33].

**Figure 2:**
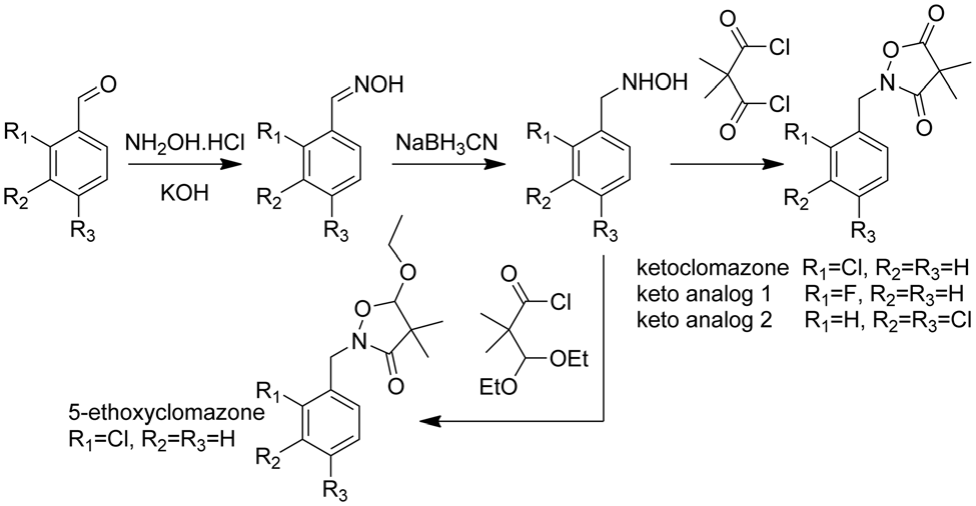
Schematics of the synthesis of 5-ethoxyclomazone, ketoclomazone and keto analogs 1 and 2.

Blockage of any of the steps preceding the formation of lycopene inhibits carotenoid synthesis. However, only the herbicide clomazone targets the MEP pathway by inhibiting DXS [34], although it does so indirectly [35]. It has been postulated that, once absorbed into the plant, clomazone is oxidized by cytochrome P450 monooxygenases (P450s) to form ketoclomazone (Figure 2), which is the putative active herbicidal form [34]. Early evidences of this requirement for metabolic activation was observed in plants treated with phorate or other P450s inhibitors being protected from the herbicidal effect of clomazone [36], [37]. The antibiotic fosmidomycin is not used as an herbicide, but this compound inhibits DXR, the second step of the MEP pathway [38], [39].

The MEP and carotenoid pathways are not present in animals, and thus, their enzymes are preferred targets for new herbicides [40]. In spite of the potential relevance of all other enzymes of this pathway, no herbicide targeting the early steps of carotenoid synthesis, other than clomazone, has been developed [41].

The emergence of resistance to herbicides is an increasing problem facing agriculture [42], [43], and there do not appear to be any herbicides with novel mechanisms of action being developed [33]. The pathways leading to carotenoids (MEP and isoprenoid pathways) offer several attractive targets for new molecules discovery efforts [44]. Indeed, the unique target sites inhibited by clomazone [37] and fosmidomycin [45] illustrate the potential benefits of developing new herbicides which interfere with the early steps of carotenoid synthesis.

One approach to discover novel inhibitors has been high throughput *in vitro* assays that are most likely to identify mechanism-based inhibitors of the various steps in the MEP pathway [46]. The aim of our research was to develop a simple, fast, and inexpensive, *in vivo* assay to identify inhibitors of the early steps in carotenoid synthesis by measuring the carbon flux through the MEP and isoprenoid pathways using phytoene as a biomarker.

## Materials and Methods

### Chemicals and supplies

Phorate, *O*,*O*-diethyl *S*-[(ethylthio]methyl) phosphorodithioate; fosmidomycin (3-(formylhydroxyamino)propyl)-phosphonic acid sodium salt; FR-900098, *p*-[3-(acetylhydroxyamino)propyl]-phosphonic acid; amitrol, 1,2,4-triazol-3-amine; dinoterb, 2-(1,1-dimethylethyl)-4,6-dinitro-phenol; endothall monohydrate, 7- oxabicyclo[2.2.1] heptane-2, 3-dicarboxylic acid monohydrate; oryzalin, 4-(dipropylamino)-3,5-dinitro-benzenesulfonamide; sulcotrione, 2-[2-chloro-4-(methylsulfonyl)benzoyl]-1,3-cyclohexanedione were purchased from Sigma-Aldrich (St. Louis, MO 63103) and dichlobenil (2,6-dichlorobenzonitrile); glufosinate-ammonium, 2-amino-4-(hydroxymethylphosphinyl)butyric acid ammonium salt were purchased from Allied-Signal Inc., (Morristown, NJ 07960).

Clomazone, 2-[(2-chlorophenyl)methyl]-4,4-dimethyl-3-isoxazolidinone; imazapyr, 2-[4,5-dihydro-4-methyl-4-(1-methylethyl)-5-oxo-1*H*-imidazol-2-yl]-3-pyridinecarboxylic acid; quinclorac, 3,7-dichloro-8-quinolinecarboxylic acid; atrazine, 6-chloro-*N*2-ethyl-*N*4-(1-methylethyl)-1,3,5-triazine-2,4-diamine; paraquat CL tetrahydrate, 1,1′-dimethyl-4,4′-bipyridinium dichloride; imazethapyr, 2-(4,5-dihydro-4-methyl-4-(1-methylethyl)-5-oxo-1*H*-imidazol-2-yl)-5-ethyl-3-pyridinecarboxylic acid; alachlor, 2-chloro-*N*-(2,6-diethylphenyl)-*N*-(methoxymethyl)-acetamide; asulam, *N*-[(4-aminophenil)sulfonyl]-carbamic acid methyl ester; sulfentrazone, *N*-[2,4-dichloro-5-[4-(difluoromethyl)-4,5-dihydro-3-methyl-5-oxo-1*H*-1,2,4-triazol-1-yl]-phenyl] methanesulfonamide; glyphosate-isopropylammonium, isopropylammonium *N*-(phosphonomethyl) glycine; diclofop-methyl, 2-[4-(2,4-dichlorophenoxy)phenoxy]-propanoic acid methyl ester were purchased from Chem-Service (West Chester, PA 19381).

Norflurazon, 4-chloro-5-(methylamino)-2-[3-(trifluoromethyl)phenyl]-3(2*H*)-pyridazinone was provided by Sandoz, Inc. Crop Protection (now Syngenta, Greensboro, NC 27419), and experimental inhibitors for some of the enzymes of the MEP pathway were provided by BASF-SE (Ludwigshafen, Germany) [41].

### Synthesis of halogenated analogs

Reagents and solvents were purchased from Sigma-Aldrich Chemical Co. (St Louis, MO, USA) and Fisher Scientific (Pittsburgh, PA, USA). NMR spectra were recorded on a Varian-Mercury-plus-400 or Varian Unity-Inova-600 spectrometer using CDCl_3_ and methanol-*d*_4_ unless otherwise stated. MS data were obtained from an Agilent Series 1100 SL equipped with an ESI source (Agilent Technologies, Palo Alto, CA, USA). Column chromatography and preparative TLC were performed on Merck silica gel 60 (230–400 mesh) and silica gel GF plates (20×20 cm, thickness 0.25 mm), respectively. General synthesis of 5-ethoxyclomazone, ketoclomazone and analogs is shown in Figure 2.

### Synthesis of halogenated-benzaldehyde oximes

Oximes of 2-chloro-, 2-fluoro, and 3,4-dichlorobenzaldehyde were prepared by the procedure reported by Zamponi et al. [47].

2-Chlorobenzaldehyde oxime: ^1^H NMR δ(CDCl3): 7.26 (1H, brt, J = 7.2 Hz), 7.31 (1H, td, J = 7.6, 1.6 Hz), 7.38 (1H, dd, J = 8.0, 1.2 Hz), 8.62 (1H, s).

2-Fluorobenzaldehyde oxime: ^1^H NMR δ(CDCl3): 7.10 (1H, brt, J = 9.2 Hz), 7.66 (1H, brt, J = 7.6 Hz), 7.37 (1H, brq, J = 7.2 Hz), 7.71 (1H, t, J = 7.6 Hz), 8.38 (1H, s).

3,4-Dichlorobenzaldehyde oxime:^ 1^H NMR δ(CDCl3): 7.40 (1H, dd, J = 8.4, 2.0 Hz), 7.46 (1H, d, J = 8.4 Hz), 7.67 (1H, d, J = 2.0 Hz), 8.01 (1H, s).

### Synthesis of halogenated N-(benzyl)hydroxylamine

A mixture of oxime (500 mg) in glacial acetic acid (10 ml) was treated with sodium cyanoborohydride (NaCNBH_3_) portion wise under stirring while maintaining the temperature below 20°C until the reaction was complete as evidenced by TLC. The reaction mixture was basified with ice-cold NaOH and extracted with ethyl acetate. The organic layer was washed with water, dried and evaporated to afford a white solid. This product was chromatographed over silica gel and eluted with ethyl acetate:hexane 3∶7 to yield a pure product which was crystallized from CHCl_2_/hexanes.

*N*(2-Chlorobenzyl)hydroxylamine: ^1^H NMR δ(CDCl3): 4.1 (2H, s), 7.21–7.26 (2H, m), 7.35–7.39 (2H, m).

*N*(2-Fluorobenzyl)hydroxylamine: ^1^H NMR δ(CDCl3): 4.06 (2H, s, CH_2_), 7.05 (1H, brt, J = 8.8 Hz), 7.13 (1H, brt, J = 7.2 Hz), 7.27 (1H, brq, J = 6.8 Hz), 7.33 (1H, brt, J = 7.6 Hz).

*N*(3,4-Dichlorobenzyl)hydroxylamine (**6**):^ 1^H NMR δ(CDCl3): 3.93 (2H, s), 7.15 (1H, dd, J = 8.0, 2.0 Hz), 7.40 (1H, d, J = 8.0 Hz), 7.44 (1H, d, J = 2.0 Hz).

### Synthesis of halogenated ketoclomazone

Dimethylmalonyl dichloride (1.25 mM) in CH_2_Cl_2_ (1 ml) was slowly added to a solution of hydroxylamine (1 mM) and triethylamine (0.3 ml) in CH_2_Cl_2_ (5 ml) at 10°C. After addition was complete, the reaction mixture was stirred for 30 min poured onto ice and extracted with CH_2_Cl_2_. The organic layer was washed with aqueous Na_2_CO_3_, 1 N HCl and saturated aqueous NaCl. The resulting solution was then dried and the gummy product obtained was chromatographed over silica gel. The product was eluted with ethyl acetate:hexane 5∶95 and crystallized in CH_2_Cl_2_/hexanes.

Ketoclomazone: ^1^H NMR δ(CDCl3): 1.44 (6H, s), 5.05 (2H, s), 7.24–7.31 (2H, m), 7.34 (1H, m), 7.39 (1H, m). 13C NMR δ(CDCl3): 21.4 (CH_3_), 41.9 (C), 47.3 (CH_2_), 127.3 (CH), 130.0 (CH), 130.1 (CH), 130.3 (CH), 131.4 (C), 133.8 (C), 172.1 (C), 173.8 (C); HRESIMS [M+H]^+^ m/z 254.0600 (calcd for (C_12_H_12_ClNO_3_+H)^+^254.0584).

2-(2-Fluorobenzyl)-4,4-dimethylisoxazolidine-3,5-dione (keto analog **1**): ^1^H NMR δ(CDCl_3_): 1.41 (6H, s), 4.98 (2H, s), 7.07 (1H, brt, J = 9.2 Hz), 7.13 (1H, brt, J = 7.6 Hz), 7.25–7.34 (2H, m). 13C NMR δ(CDCl3): 21.2 (CH_3_), 41.8 (C), 47.3 (CH_2_, *J_CF_* = 4.4 Hz), 115.8 (CH, *J_CF_* = 21.3 Hz), 120.7 (CH, *J_CF_* = 14.0 Hz), 124.5 (CH, *J_CF_* = 3.6 Hz), 130.5 (CH), 130.6 (CH, *J_CF_* = 5.9 Hz), 160.9 (C, *J_CF_* = 248 Hz), 172.4 (C), 173.6 (C); HRESIMS [M+H]^+^ m/z 237.0811 (calcd for (C_12_H_12_FNO_3_+H)^+^237.0801).

2-(3,4-Dichlorobenzyl)-4,4-dimethylisoxazolidine-3,5-dione (keto analog **2**): ^1^H NMR δ(CDCl3): 1.40 (6H, s), 4.83 (2H, s), 7.17 (1H, dd, J = 8.0, 2.0 Hz), 7.41 (1H, brs), 7.42 (1H, d, J = 2 Hz), 7.43 (1H, d, J = 8.0 Hz). 13C NMR δ(CDCl3): 21.3 (CH_3_), 42.0 (C), 48.8 (CH_2_), 128.0 (CH), 130.6 (CH), 131.1 (CH), 133.1 (C), 133.2 (C), 134.0 (C), 172.9 (C), 173.5 (C); HRESIMS [M+H]^+^ m/z 288.0207 (calcd for (C_12_H_11_Cl_2_NO_3_+H)^+^288.0194).

### Synthesis of 5-ethoxyclomazone

A mixture of ethyl 3,3-diethoxy-2,2-dimethylpropionate [48] and KOH (2.2 g) in 10% aqueous ethanol (40 ml) was refluxed for 2 hours and the solvent was evaporated under vacuum. The residue was dissolved in water (40 ml), neutralized with succinic acid and extracted with CH_2_Cl_2_. The organic layer was washed with saturated aqueous NaCl, dried over anhydrous Na_2_SO_4_, and evaporated to give 3-diethoxy-2-dimethylpropanoic acid as a thick oil. Upon storage at 4°C this oil formed a white crystalline solid.

1H-NMR (400 MHz) δ 1.17 (6H, s, CH_3_), 1.17 (3H, t, J = 7.0 Hz, CH3), 3.55 (2H, dq, *J* = 16.0, 7.2 Hz, CH_2_), 3.82 (2H, dq, *J* = 16.0, 7.2 Hz, CH_2_), 4.54 (1H, s, CH); 13C-NMR (100 MHz) δ 15.3 (CH_3_), 19.6 (CH_3_), 48.4 (C), 66.5 (CH_2_), 107.3 (CH), 181.9 (CO); HRESIMS [M+Na]^+^ m/z 213.1099 (calcd for (C_9_H_18_O_4_+Na)^+^213.1103).

Oxalyl chloride (260 mg, 4 mm) was added to a solution of 3-diethoxy-2-dimethylpropanoic acid (380 mg) in toluene (3 ml) and the reaction mixture was stirred at 60°C for 30 minutes. The solvent was evaporated under vacuum to afford oil. This oil was dissolved in toluene 5 ml and evaporated under vacuum. This product was used in the next reaction immediately without further purification.

Triethylamine (0.5 ml) and 2-chloro-*N*-hydroxybenzylamine (200 mg 1.27 mm) were added sequentially to a solution of 3-diethoxy-2-dimethylpropanoic acid chloride (2 mm) in CH_2_Cl_2_ (4 ml) at 0°C under stirring. The reaction mixture was stirred over night at room temperature and partitioned between water and CH_2_Cl_2_. The organic layer was washed with water, dried over Na_2_SO_4_, and evaporated. The products were chromatographed on silica gel and elution with ethyl acetate/hexane (1∶99) gave the least polar product, 5-ethoxyclomazone, as an colorless oil (32 mg).

1H-NMR (400 MHz) δ 1.06 (3H, t, *J* = 7.2 Hz, CH_3_), 1.16 (3H, s, CH_3_), 1.16 (3H, s, CH_3_), 1.24 (3H, s, CH_3_), 3.38 (1H, dq, *J* = 16.0, 7.2 Hz, CH) 3.53 (1H, dq, *J* = 16.0, 7.2 Hz, CH), 4.73 (1H, d, *J* = 16.8 Hz, CH_2_), 4.83 (1H, s, CH), 4.73 (1H, d, *J* = 16.8 Hz, CH), 4.89 (1H, d, *J* = 16.8 Hz, CH), 7.16–7.25 (2H, m, CH), 7.29–7.35 (2H, m, CH); 13C-NMR (100 MHz) δ 14.8 (CH_3_), 16.6 (CH_3_), 22.4 (CH_3_), 46.1 (CH_2_), 46.3 (C), 64.1 (CH_2_), 108.0 (CH), 126.8 (CH), 129.0 (CH), 129.2 (CH), 129.4 (CH), 132.9 (C), 133.2 (C), 172.8 (CO); HRESIMS [M+H]^+^ m/z 283.0969 (calcd for (C_14_H_18_ClNO_3_+H)^+^283.0975).

### Plant material

Barley (*Hordeum vulgare* L.) seeds were purchased from Johnny’s Selected Seeds (Waterville, Maine 04903). Seeds were sown in moist commercial Metromix potting soil and grown either in a dark growth chamber set at 25°C or in the greenhouse under natural light for 4 days.

### Bioassays

Approximately 0.1 g of fresh young barley leaves were weighed, cut in 3 mm sections with a razor blade, and incubated (60×15 mm Petri dishes) in 5 ml of 5 mM 2-[*N*-morpholino]ethanesulfonic acid buffer (MES, pH 6.5) containing 200 µM of norflurazon for 24 h in a growth chamber with the 16/8 light/dark cycle at 25°C. The herbicides and other test compounds (see section *Chemicals and Supplies*) were tested either at a fixed concentration or at different concentrations(dose-response curves ranged from 0.1 to 100 µM inhibitor in acetone). Control tissues were exposed to the same amount of acetone as the treated tissues but without the test compounds. All experiments had 3–5 replicates and were repeated in time. The effect of 50 µM phorate, a cytochrome P450 monooxygenase inhibitor, was tested in some of the assays with clomazone to determine the requirement for the metabolic activation of this herbicide.

The usefulness of this bioassay in identifying potential novel inhibitors of the early steps of carotenoid biosynthesis was tested with a number of experimental compounds provided by BASF or synthesized in our laboratory. These compounds were tested at a 100 µM final concentration on greening etiolated barley leaf cuttings and their activity is expressed as inhibition of phytoene accumulation relative to the amount of phytoene accumulating in the norflurazon alone treatment. Finally, the specificity of this assay was evaluated by testing representative compounds inhibiting all the known target sites of commercial herbicides. These compounds were tested at a 100 µM final concentration on etiolated barley leaf cutting either exposed to light or maintained in total darkness for 24 h. Herbicides causing a 10% or less reduction of phytoene level were considered not active. Those causing 10 to 50% inhibition and those causing more than 50% inhibition were considered slightly and highly active, respectively.

### Phytoene extraction and determination

Phytoene was extracted and quantified according to a protocol modified from Sprecher et al. [49] as described in Dayan et al. [50]. After 24 h incubation, the barley leaf samples were homogenized (Polytron, PT 3300) in 3 ml of 6% KOH in methanol (w/v), stored for 15 min at room temperature (RT) and then centrifuged for 5 min at 1,300 *g* (Sorvall Swinging Bucket SH-3000 rotor). The supernatant was transferred into new tubes and mixed with 3 ml of petroleum ether (Acros, Fair Lawn, NJ, boiling range 80–110°C). Saturated NaCl solution was added (1.5 ml), mixed and centrifuged for 10 min at 1,300 *g*. A clear partition is formed by centrifugation and an aliquot (1.250 ml) of the epiphase was transferred to disposable cuvettes (methacrylate) followed by UV-spectrophotometric (Shimadzu, UV-3101PC) measurements at 287 nm. All manipulations were performed in a dark room under green light. The phytoene content was calculated by its extinction coefficient (ε) of 1108 mM cm^−1^ and expressed as µg g^−1^ fresh weight (FW) according to equation 1:
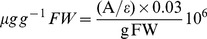
(1)


### Statistical Analysis

Phytoene accumulation was plotted against inhibitor concentrations to generate dose-response curves. Data were analyzed by a four-parameters log-logistic model [51] using R software (version 2.15.2, R Foundation for Statistical Computing, Vienna, Austria) with the drc module [52]. Means and standard deviations were obtained using the raw data and the half-maximal inhibitory response (I_50_) was defined as the concentration at which this accumulation was inhibited by 50% compared with controls. I_50_ values were obtained from the parameters in the regression curves. Graphs were generated with Sigma Plot (version 11, Systat Software Inc., San Jose, CA, USA). Means were separated with the Duncan multiple range test at *P* = 0.05 using the Agricolae module [53].

The quality of the assay was determined by calculating the Z′ factor using equation 2 according to Zhang et al. [54]

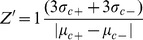
(2)where 3σ_c+_ = 3 standard deviations of positive control, 3σ_c–_ = 3 standard deviations of negative control, µ_c+_ = mean of positive control and µ_c–_ = mean of negative control. The utilization of the 3 standard deviations ensures 99.73% confidence limit. The Z′ of the phytoene accumulation assay is based on the measurements of 33 positive controls and 33 negative controls.

## Results and Discussion

### Accumulation of phytoene over time

**Figure 3:**
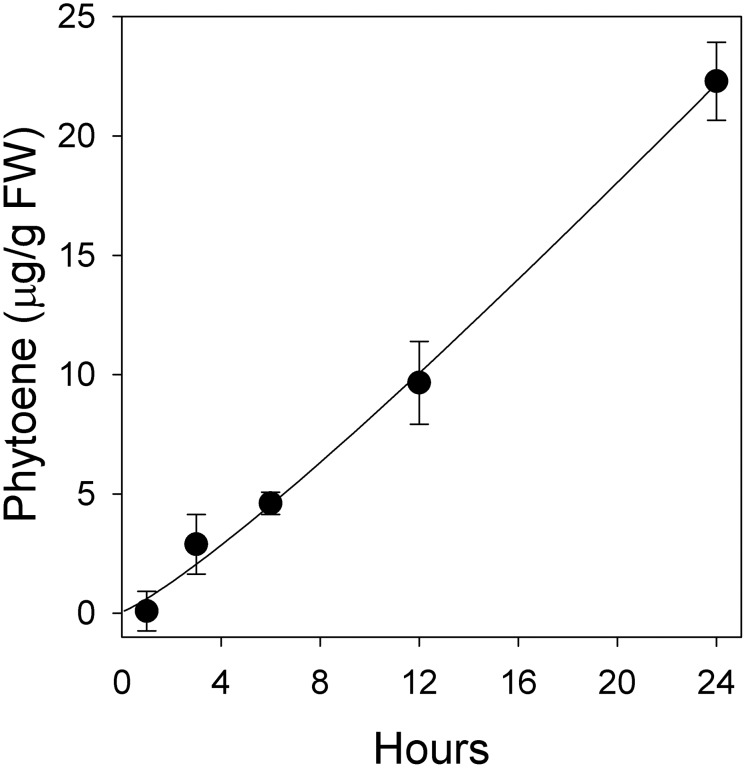
Time-dependent phytoene accumulation in barley (*Hordeum vulgare* L.) exposed to 200 µM norflurazon. Data represent means of three replications with standard deviation.

This simple assay relies on the premise that the accumulation of phytoene resulting from the inhibition of phytoene desaturase by norflurazon is a reflection of the carbon flow through the MEP pathway. Incubation of barley leaves floating on a medium supplemented with 200 µM norflurazon for 24 h caused a time-dependent linear accumulation of phytoene (Figure 3). Therefore, any compound reducing the level of phytoene accumulation during this assay is likely to inhibit one of the many enzymatic steps leading to phytoene synthesis (Figure 1). It has been suggested that inhibiting the MEP pathway of plants could be useful in the search of novel herbicides [55] and the bioassay described herein may be a useful new tool to discover such compounds.

Barley was selected because its small seeds germinate quickly in the dark and contain highly active MEP and carotenoid pathways during its light-induced thylakoid formation, in the transition of etioplasts to chloroplasts during the greening process [56]. Additionally, it is highly sensitive to clomazone [57], which is important because some plants do not metabolize clomazone to ketoclomazone (the putative active form) very rapidly, and their inhibitory effects may not be detected during the time-span of this experiment.

### Effect of clomazone, ketoclomazone, and 5-ethoxyclomazone

Clomazone is the only commercial herbicide known to inhibit carotenoid synthesis upstream from phytoene desaturase. Actually, clomazone is inactive, but its metabolite ketoclomazone inhibits DXS [34], [56], the thiamine diphosphate-dependent enzyme that catalyzes the first step in the MEP pathway [58].

**Figure 4:**
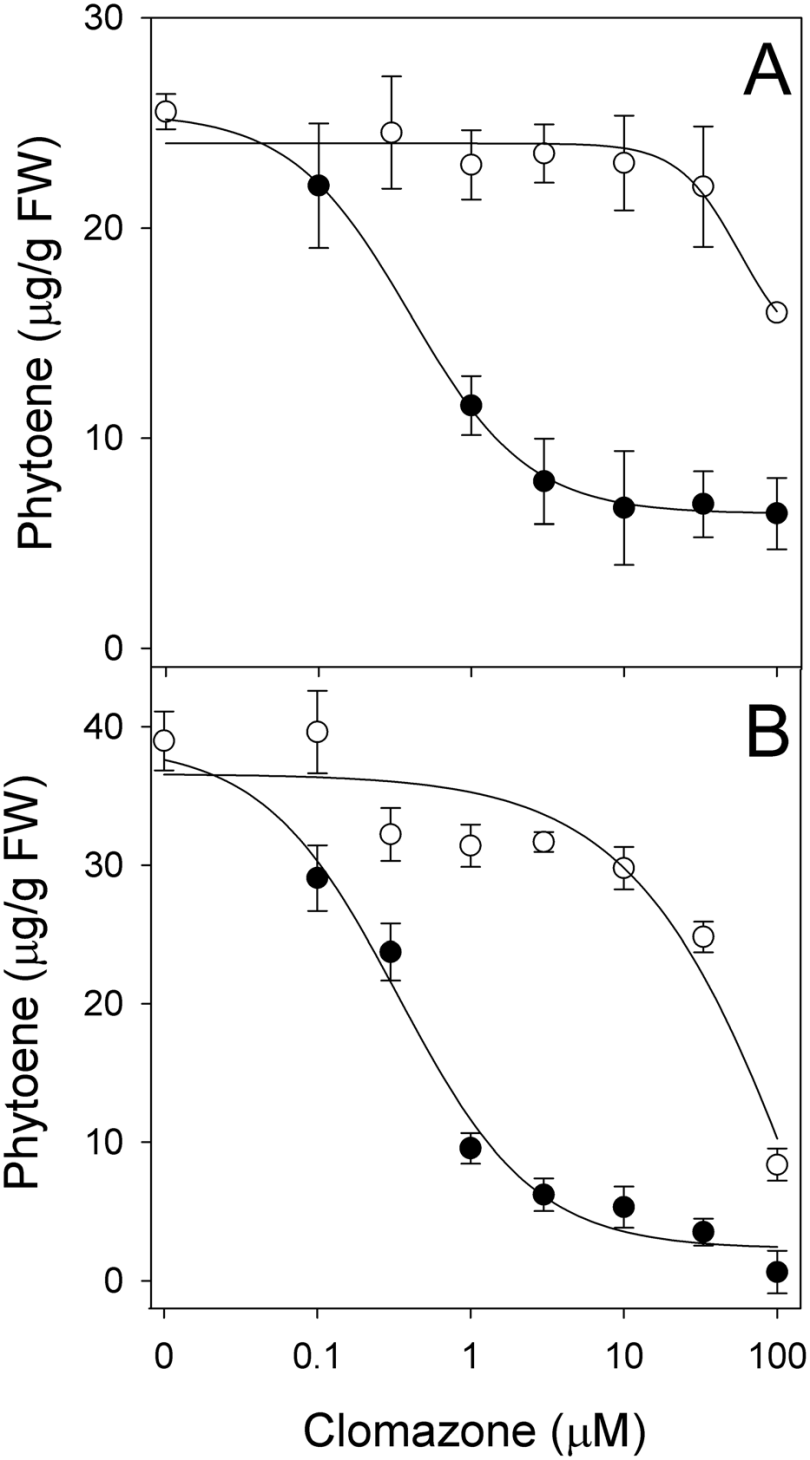
Dose-response curves showing the effect of the herbicide clomazone with (○) and without (•) phorate on phytoene accumulation induced by 200 µM norflurazon. (A) Green and (B) greening etiolated young barley leaves. Data represent means of three replications with standard deviation.

In our simple barley leaf cutting assay, clomazone inhibited phytoene accumulation in a dose-dependent manner that illustrates the inhibition of carbon flow into the MEP pathway in both green (Figure 4A) and greening etiolated tissues (Figure 4B). Clomazone had an I_50_ for inhibition of phytoene accumulation of 0.6±0.16 and 0.33±0.05 µM in green and greening tissues, respectively. Sandmann and Böger [30] reported an I_50_ value for clomazone of less than 15 µM for inhibition of phytoene and phytol biosynthesis in spinach extracts, suggesting that our *in vivo* assay may be more sensitive as it allows for the metabolic activation of clomazone [36], [37].

The requirement for metabolic activation of clomazone was confirmed by repeating the same dose-response curves in the presence of 50 µM phorate. Phorate is an organophosphate insecticide that inhibits cytochrome P450 monooxygenases in plants [59]. In some species, clomazone is rapidly metabolized by P450s [60]–[62] and phorate can protect plants against the phytotoxic effect of clomazone by preventing this metabolic activation [36], [63].

The addition of 50 µM phorate to the solution prevented clomazone from inhibiting phytoene accumulation in both green and greening etiolated, with I_50_>100 µM (Figures 4 A and B). These results are consistent with previous studies indicating that clomazone must be metabolically activated by P450s and that phorate can abolish its herbicidal activity [36], [63].

In plants, metabolism of clomazone follows a couple of pathways. One route involves a *N*-dealkylation step yielding 2-chlorobenzyl alcohol. The other route follows the progressive oxidation of carbon 5 to yield 5-hydroxyclomazone and ultimately ketoclomazone [60], [62]. In both of these studies, 5-hydroxyclomazone and ketoclomazone were present in equivalent amounts, suggesting that the conversion of clomazone to ketoclomazone is not instantaneous. While ketoclomazone has been proposed as the active form responsible for the herbicidal activity of clomazone [34], [56], early work on the development of clomazone by FMC (Philadelphia, PA USA) also reported that 5-hydroxyclomazone and several 5-alkoxy derivatives were also potent herbicides [64]. It is postulated that all these 5-hydroxy derivatives are further metabolized into ketoclomazone.

**Figure 5:**
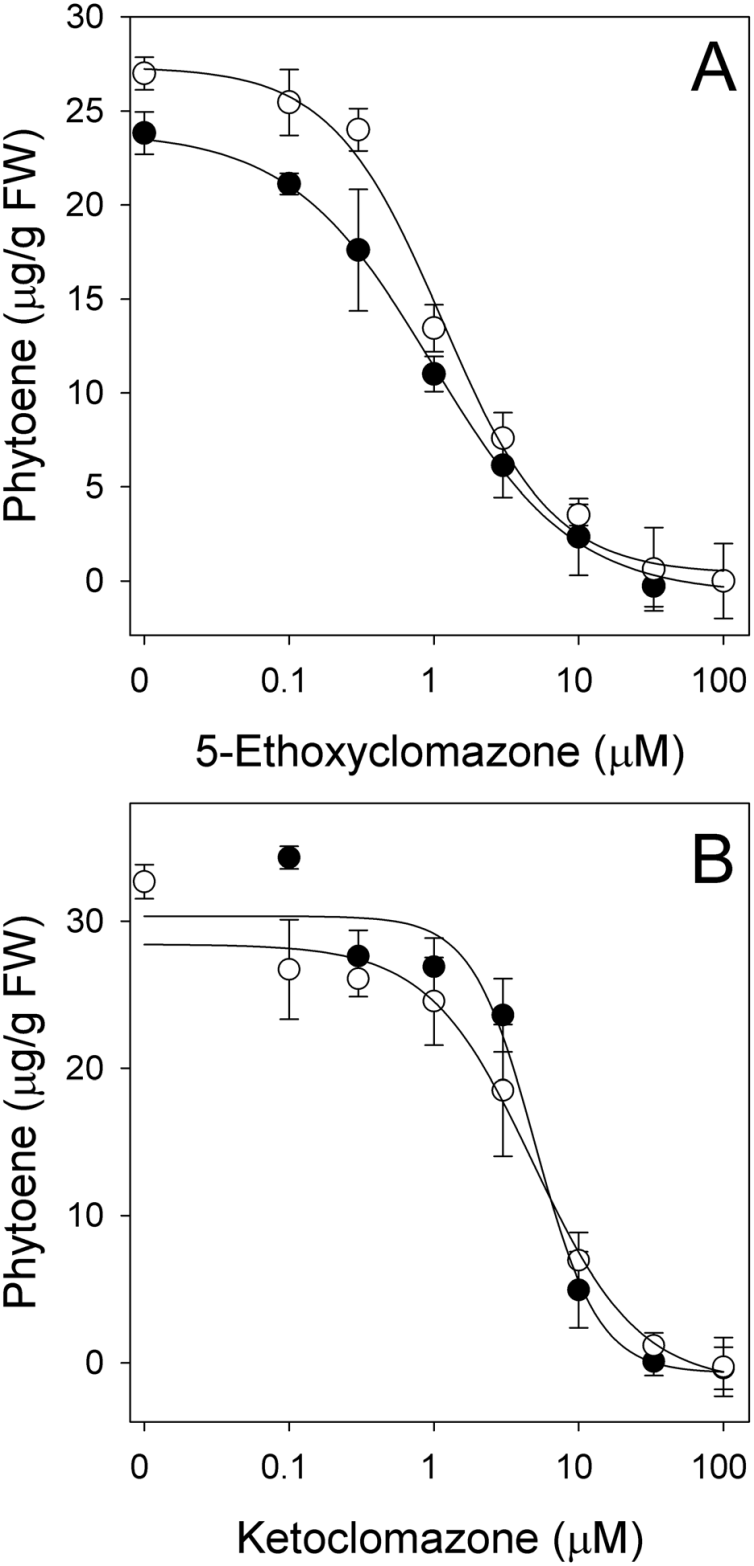
Dose-response curves of (A) 5-ethoxyclomazone and (B) ketoclomazone with (○) and without (•) phorate on greening etiolated barley leaves. Phytoene was caused to accumulate by the presence of 200 µM norflurazon. Data represent means of three replications with standard deviation.

When tested in our bioassay, 5-ethoxyclomazone was as active as clomazone, and phorate did not negatively affect that activity (Figure 5A). Although still very active, ketoclomazone was 16 times less active in greening tissues than clomazone, with I_50_ values of 5 µM (Figure 5B). This was unexpected since clomazone is a proherbicide and appears to be bioactivated by P450s (Figure 4). Phorate did not affect the activity of ketoclomazone, suggesting that it is not subject to further oxidation by P450s (Figure 5B). Similar results were obtained in green tissues (Figure S1).

From a metabolic perspective, the pattern observed with clomazone, 5-ethoxyclomazone and ketoclomazone, and the effect of phorate on the activity of these compounds is particularly informative. Clomazone is a potent *in vivo* inhibitor of phytoene accumulation but phorate greatly reduces this activity, confirming that clomazone is not herbicidal and that one of its oxidized metabolites must be very active, causing inhibition of the carbon flow at submicromolar concentrations. 5-Ethoxyclomazone is as active as the commercial herbicide. On the other hand, the activity of ketoclomazone was lower than that of clomazone, and phorate had no effect on the herbicidal activity of ketoclomazone, suggesting that either the uptake of ketoclomazone was limiting, or that another intermediate is the active form of the herbicide.

Clomazone, 5-hydroxyclomazone and a series of acylated 5-hydroxy derivatives do not inhibit purified plant DXS, whereas ketoclomazone is highly active, with an *I*_50_ value of 80 nM [41]. Additionally, the *seco*-ketoclomazone analog with a 3-[methyl(hydroxy)amino]-2,2-dimethyl-3-oxopropanoic acid side chain, which is a known plant metabolite of clomazone [60], [62], was recently reported as a potent inhibitor of *Haemophilus influenza* DXS [65]. Analysis of the structure-activity relationship of the compounds tested in that study highlighted the requirement for the hydroxamate, the 2,2-dimethylmethylidene, and the carboxylic functional groups. While this *seco-*ketoclomazone analog has structural features reminiscent of a putative transition state intermediate analog, neither the exact form of the clomazone pharmacophore responsible for inhibition of DXS nor its binding to the enzyme is known.

### Validity of the bioassay with inhibitors of early steps of the MEP pathway

Keto analogs **1** and **2** are structurally related to ketoclomazone with simple changes in the halogen substitutions on the phenyl ring (Figure 2). In this study, keto analog **1** was a weak inhibitor of phytoene accumulation, with an I_50_ value of 22 µM on greening etiolated tissue (Figure S2A). Its activity on green tissue was even lower, with an I_50_ value of 52 µM (Figure S2B). However, the structural similarity between ketoclomazone and keto analog **1** suggests that this new compound may inhibit DXS. Keto analog **2** did not affect phytoene accumulation (data not shown), suggesting that the position of the halogen on the phenyl ring has a significant effect on the activity of ketoclomazone.

The first compound described to inhibit the MEP pathway was fosmidomycin, also known as FR-31564 and 3-(*N*-formyl-*N*-hydroxyamino) propylphosphonic acid [66], [67]. This compound prevents the conversion of labeled DOXP into carotenoids in th*e Capsicum* chromoplast system [68] by inhibiting DXR (Figure 1) [66]. Since then, a considerable number of fosmidomycin derivatives have been synthesized to identify new DXR inhibitors [69], [70].

**Figure 6:**
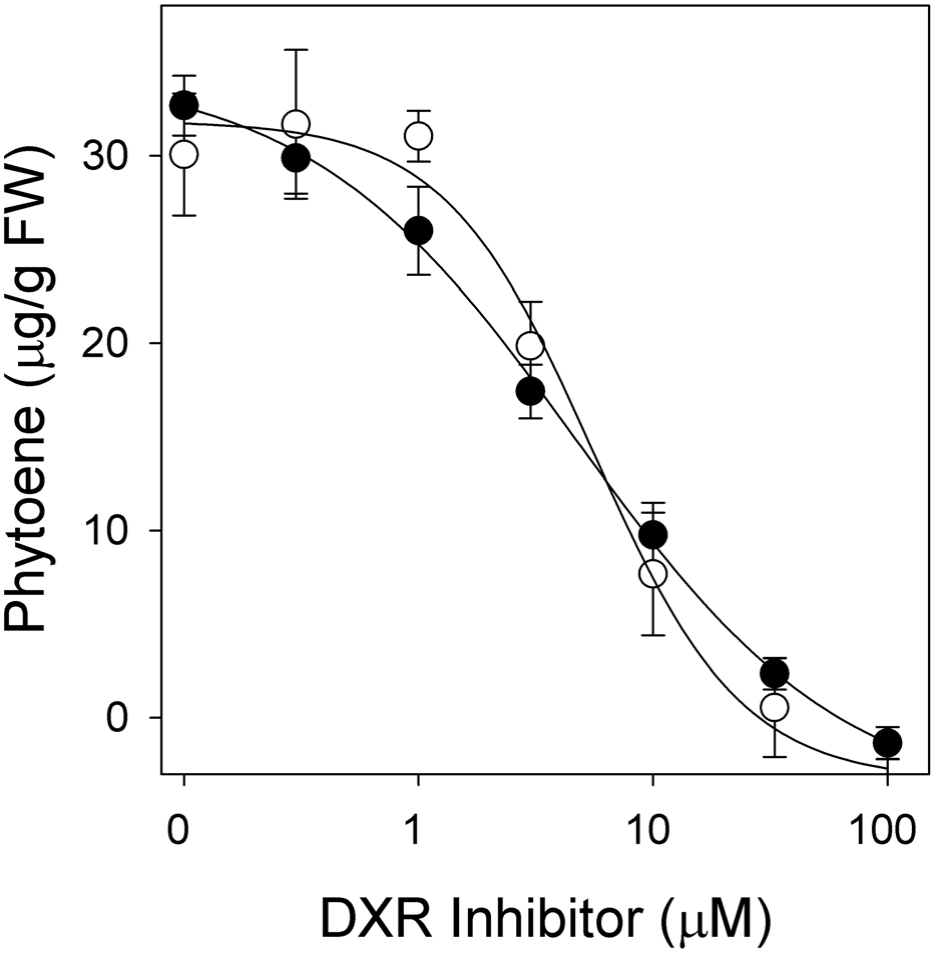
Dose-response curves of the DXR inhibitors fosmidomycin (•)and FR-900098 (○) on greening etiolated barley leaves. Phytoene accumulation was induced in the presence of 200 µM norflurazon. Data represent means of three replications with standard deviation.

In our study, fosmidomycin and its structural analog FR-900098 inhibited phytoene accumulation in a dose-dependent manner with I_50_ values of 5 and 5.5 µM for greening etiolated, respectively (Figure 6). Similar activity was observed on green barley leaves, with I_50_ values of 11 and 17.5 µM for fosmidomycin and FR-900098, respectively (Figure S3).

Interest in inhibitors of the MEP pathway has led a research group at BASF-SE (Ludwigshafen, Germany) to screen for new herbicides targeting this pathway in target-based high throughput *in vitro* assays [41]. Eleven of these experimental compounds were tested in our *in vivo* bioassay.

None of these compounds were very active in our *in vivo* bioassay (Table 1). The most active cluster were **BASF 11** and **BASF 12**, the azolopyrimidine inhibitors of 2*C*-methyl-d-erythritol 4-phosphate cytidyltransferase (IspD), the third step on the MEP pathway (Figure 1), with 63.6% and 61.3% inhibition of phytoene accumulation, respectively (Table 1). Interestingly, these compounds were the most potent MEP inhibitors generated by BASF with *in vitro* I_50_ values against IspD activity at 140 nM and 35 nM, respectively [41]. IspD activity has been validated by antisense experiments to be essential to plant survival and inhibitors of this enzyme were anticipated to be potentially herbicidal [71], [72]. All of the other BASF compounds, including the putative inhibitors of 4-diphosphocytidyl-2*C*-methyl-d-erythritol kinase (ispE), had *in vitro* activity in the micromolar range [41] and were not very active in the barley bioassay (Table 1). The difference between the activity reported on the target sites and in the barley leaf bioassay may be due to a number of factors. There is often no correlation between the *in vitro* activity of experimental compounds and their performance as herbicides *in vivo* because their physicochemical properties may be less than ideal for uptake and translocation [33]. Metabolic degradation of the compounds may also play a role.

**Table 1:** Effect of BASF experimental compounds on phytoene accumulation in the presence of 200 µM norflurazon.

MEP target site		BASF	Inhibition^b^	
		#^a^	(%)	
DXS				
1-Deoxyxylulose-5-phosphate synthase				
	3		11.1	BC
	4		15.6	BC
	5		27.2	CD
	6		44.4	DE
DXR				
1-Deoxyxylulose-5-phosphate reductoisomerase				
	7		(117.7)^c^	A
	8		40.3	DE
IspD				
2*C*–Methyl-d-erythritol 4-phosphate cytidyltransferase				
	9		20.6	BCD
	10		26.0	CD
	11		63.6	E
	12		61.3	E
IspE				
4-Diphosphocytidyl-2*C*-methyl-d-erythritol kinase				
	13		28.8	CD

Inhibition of phytoene accumulation by compound treatment was expressed in percentage of inhibition related to maximum accumulation in control assays. All compounds were tested at 100 µM.

a Numbering corresponds to structures in Witschel et al. (2013) [41].

b Means values followed by the same letter do not differ significantly at the 5% level by Duncan’s multiple range test.

c Caused greater phytoene accumulation.

### Selectivity of the bioassay

Herbicides have unique affinities for their respective molecular target sites within important plant biochemical pathways and/or physiological processes [35], [73]. To test the selectivity of the bioassay to identify inhibitors of the early steps of carotenoid synthesis, herbicides representative of all known modes of action were selected to survey their effects on phytoene accumulation (Table 2). All of the compounds were tested at 100 µM to determine whether they would be detected as false positives in a screening program.

**Table 2:** Effect of herbicides with different modes of action on phytoene accumulation in the presence of 200 µM norflurazon.

WSSA	Compound^a^	MOA	Inhibition^b^	
Class			Light	Dark
A	Diclofop	Acetyl-CoA carboxylase	+	–
B	Imazethapyr	Acetolactate synthase	–	–
C1	Atrazine	Photosystem II	–	–
D	Paraquat	Photosystem I electron diverter	++	–
E	Sulfentrazone	Protoporphyrinogen oxidase	++	+
F2	Sulcotrione	*p*-Hydroxyphenylpyruvate dioxygenase	+	–
F4	Clomazone	Deoxyxylulose-5-phosphate synthase	++	++
G	Glyphosate	Enolpyruvylshikimate synthase	+	–
H	Glufosinate	Glutamine synthetase	–	–
I	Asulam	Dihydropteroate synthase	+	–
K1	Oryzalin	Tubulin	–	–
K3	Alachlor	Very long chain fatty acid elongases	–	–
L	Dichlobenil	Cellulose synthase	–	–
M	Dinoterb	Oxidative phosphorylation uncoupler	++	++
NC	Endothall	Serine/threonine protein phosphatase	++	+
O	Quinclorac	Synthetic auxin	–	–

The bioassay was performed in three replications of each treatment.

a All compounds were tested at 100 µM to determine whether they would be detected as false positives in a high throughput screening program.

b no inhibition (0–10%) (–), slight inhibition (10 to 50%) (+) and strong inhibition (50 to 100%) (++).

Most of the herbicides targeting enzymes in pathways unrelated to carotenoid synthesis had either little or no effect on the carbon flow into phytoene (−/+). The slight inhibition caused by glyphosate may be a reflection of the secondary effect of this herbicide on the carotenoid pathway. Studies to understand the mechanism of vacuolar sequestration involved in resistance to glyphosate revealed that 2-*C*-methyl-*d*-erythritol-2,4-cyclopyrophosphate, the last intermediate in the MEP pathway, accumulated in the presence of this herbicide [74], [75].

Activities of a large number of herbicide families are directly or indirectly influenced by light. Herbicide mechanisms that are light-dependent or enhanced by light fall into several different categories [76]. Herbicides with the strongest indirect inhibitory effects (++) on phytoene accumulation were those generating reactive oxygen species (ROS) that lead to lipid peroxidation via light-dependent processes (e.g., inhibitors of PPO, electron diverters from photosystem I) (Table 2). This effect is most likely due to membrane degradation affecting all the biochemical process in the damaged tissues. In most cases, this interference could be alleviated by performing the assays in the dark.

To explore the impact of oxidative stress on the expression of MEP-pathway enzymes, Chang [12] exposed *Catharanthus roseus* leaf discs to a 0.5 µM paraquat solution. This treatment resulted in the bleaching within 10 h of exposure, whereas control treatment did not show any bleaching over a 30 h period. The paraquat treatment also caused a strong induction of *DXS* transcripts, suggesting deregulation of the MEP pathway.

The precise relationship between the effect of the herbicide dinoterb, a synthetic phenol that is no longer used as a herbicide, on phytoene accumulation and its mechanism of action is not well understood but this compound is a strong generator of ROS that destabilizes membranes under both light and dark conditions. Dinoterb also uncouples oxidative phosphorylation, which may reduce the endogenous levels of ATP, thereby inhibiting some of the steps in the MEP pathway (Figure 1). Endothall, an inhibitor of serine/threonine protein phosphatases, also caused a reduction of phytoene accumulation under both light and dark conditions, but the biochemical basis for this effect is unknown.

## Conclusions

The simple *in vivo* bioassay developed in this study proved to be an efficient and inexpensive screening method for putative novel inhibitors of the MEP and terpenoid pathways preceding carotenoid synthesis. The method permitted determination of carbon flow through this pathway with accuracy, reproducibility, and minimal sample consumption. The assay appears to be robust in terms of detecting the inhibitory activity of compounds that target the early steps of carotenoid synthesis. The quality of an assay is most commonly assessed by calculating the Z′ factor [77]. It is generally accepted that assays with 0.5<Z′<1 have good separation of the distributions and are excellent assays. The assay developed in this study has a Z′ factor of 0.584 (Figure S4), which indicates that is likely to successfully identify active compounds [54]. Additionally, it would easily complement enzyme-based high-throughput screenings and evaluate the *in vivo* activity potential performance of inhibitors identified in the *in vitro* assay, without requiring greenhouse tests. The sensitivity of the bioassay to compounds producing reactive oxygen species may be a limitation.

## Supporting Information

Figure S1 **Dose-response curves of ketoclomazone (a clomazone metabolite) with (○) and without (•) phorate in green barley leaves.** Phytoene was caused to accumulate by the presence of 200 µM norflurazon. Data represent means of three replications with standard deviation.
(DOC)

Figure S2 **Dose-response to the keto analog 1 on greening etiolated (A) and green (B) young barley leaves (•).** Data represent means of three replications with standard deviation.
(DOC)

Figure S3 **Dose-response curves of the DXR inhibitors fosmidomycin (○) and FR-900098 (•) on green young barley leaves.** Phytoene accumulation was induced in the presence of 200 µM norflurazon. Data represent means of three replications with standard deviation.
(DOC)

Figure S4 **Calculation of the Z′ factor of the bioassay.**
(DOCX)
