# Supplementary material for: Novel Bioassay for the Discovery of Inhibitors of the 2-C-Methyl-D-erythritol 4-Phosphate (MEP) and Terpenoid Pathways Leading to Carotenoid Biosynthesis
Source: PLoS One. 2014 Jul 31;9(7):e103704. doi: 10.1371/journal.pone.0103704 (PMC4117606; doi:10.1371/journal.pone.0103704)
Supplement: Figure S4 — Calculation of the Z′ factor of the bioassay. (DOCX) [file pone.0103704.s004.docx]

**Figure S4.** **Calculation of the Z' factor of the bioassay.**

Positive control = maximum phytoene accumulation

Negative control = maximum inhibition of phytoene accumulation

The Z' of the phytoene accumulation assay is based on the measurements of 33 positive controls and 33 negative controls.

$$Z^{'}=1-\frac{\left( 3\text{c+}+3\text{c-} \right)}{\left| \text{c+}- \text{c-} \right|}$$

where σ_c+_=8.173412, σ_c-_=5.96978, μ_c+_=-1.24248 and μ_c-_=100

$$Z^{'}=1-\frac{\left( 3x8.173412+3x5.860478 \right)}{\left| (-1.24248)-100 \right|}$$

Therefore Z' = 0.58415
