# Supplementary material for: Novel Bioassay for the Discovery of Inhibitors of the 2-C-Methyl-D-erythritol 4-Phosphate (MEP) and Terpenoid Pathways Leading to Carotenoid Biosynthesis
Source: PLoS One. 2014 Jul 31;9(7):e103704. doi: 10.1371/journal.pone.0103704 (PMC4117606; doi:10.1371/journal.pone.0103704)
Supplement: Supplementary file 2 [file pone.0103704.s001.doc]

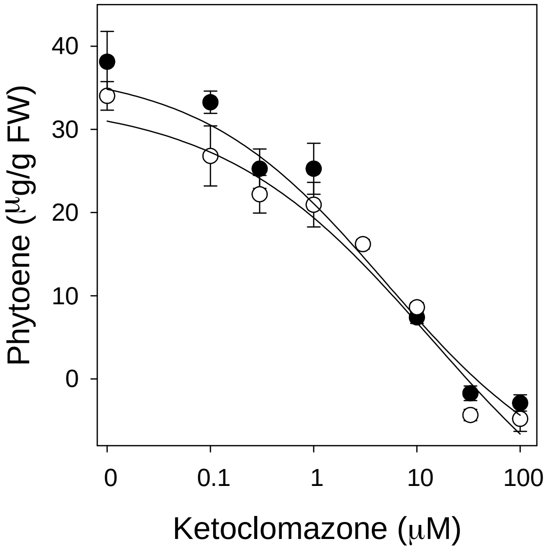
40
30
20
10
0
Phytoene (μg/g FW)
0
0.1
1
10
100
Ketoclomazone (μM)
